# Machine Learning-Based IoT-Botnet Attack Detection with Sequential Architecture †

**DOI:** 10.3390/s20164372

**Published:** 2020-08-05

**Authors:** Yan Naung Soe, Yaokai Feng, Paulus Insap Santosa, Rudy Hartanto, Kouichi Sakurai

**Affiliations:** 1 Department of Informatics, Kyushu University, Fukuoka 819-0395, Japan; sakurai@inf.kyushu-u.ac.jp; 2 Department of Electrical and Information Engineering, Universitas Gadjah Mada, Yogyakarta 55281, Indonesia; insap@ugm.ac.id (P.I.S.); rudy@ugm.ac.id (R.H.); 3 Department of Advanced Information Technology, Kyushu University, Fukuoka 819-0395, Japan

**Keywords:** IoT, botnets, machine learning, IDS, feature selection

## Abstract

With the rapid development and popularization of Internet of Things (IoT) devices, an increasing number of cyber-attacks are targeting such devices. It was said that most of the attacks in IoT environments are botnet-based attacks. Many security weaknesses still exist on the IoT devices because most of them have not enough memory and computational resource for robust security mechanisms. Moreover, many existing rule-based detection systems can be circumvented by attackers. In this study, we proposed a machine learning (ML)-based botnet attack detection framework with sequential detection architecture. An efficient feature selection approach is adopted to implement a lightweight detection system with a high performance. The overall detection performance achieves around 99% for the botnet attack detection using three different ML algorithms, including artificial neural network (ANN), J48 decision tree, and Naïve Bayes. The experiment result indicates that the proposed architecture can effectively detect botnet-based attacks, and also can be extended with corresponding sub-engines for new kinds of attacks.

## 1. Introduction

### 1.1. Background and Motivation

The Internet of Things (IoT) devices have been rapidly developing in recent years, and it makes our daily activities more convenient. The Cisco report [1] expressed that mobile traffic will grow up to 49 exabytes per month by 2021. The average of IoT devices was attacked once every two minutes, according to the Symantec report [2]. According to the Kaspersky report [3], they could collect 121,588 malware samples for IoT devices in 2018, around four times more than in 2017. Many of such malware is strong and dangerous to IoT devices. For instance, BASHLILE and Mirai are two typical IoT malware, and they infected many IoT devices, including making them accessible with vulnerabilities and known authentication credentials. BASHLILE, is also called Gafgyt, was reported in 2015; its successor, called Mirai, was reported in 2016. The infected devices will scan to identify the next possible vulnerable device and report to the central database [4]. About 2.5 million IoT devices were reportedly infected with Mirai in 2016 [4].

Many studies are trying to protect against these botnet attacks on the IoT environment. However, there are many gaps still existing to develop an effective detection mechanism. An intrusion detection system (IDS) is one of the efficient ways to deal with attacks. However, the traditional IDSs are often not able to be deployed for the IoT environments due to the resource constraint problem of these devices. The complex cryptographic mechanisms cannot be embedded in many IoT devices either for the same reason. There are mainly two kinds of IDSs: the anomaly and misuse approaches. The misuse-based, also called the signature-based, approach, is based on the attacks’ signatures, and they can also be found in most public IDSs, specifically Snort [5] and Suricata [6]. Formally, the attacker can easily circumvent the signature-based approaches, and these mechanisms cannot guarantee to detect the unknown attacks and the variances of known attacks. The anomaly-based systems are based on normal data and can support to identify the unknown attacks. However, the different nature of IoT devices is being faced with the difficulty of collecting common normal data. The machine learning-based detection can guarantee detection of not only the known attacks and their variances. Therefore, we proposed a machine learning-based botnet attack detection architecture. We also adopted a feature selection method to reduce the demand for processing resources for performing the detection system on resource constraint devices. The experiment results indicate that the detection accuracy of our proposed system is high enough to detect the botnet attacks. Moreover, it can support the extension for detecting the new distinct kinds of attacks.

### 1.2. Challenging Issues

The traditional attack detection systems cannot be competently relocated in the IoT environments because of the different nature of such devices, and the diverse architecture of the underlying network methodologies with the conventional network. Additionally, the possible attacks can be distinct from the attacks that are found on the traditional network devices. The heavyweight encryption methods cannot be deployed on these resource constraint devices. On the other side, the IoT devices become very cheap to set up for personal usages, like in small business and smart home appliances. The attackers were launching the attacks to the victim nodes after infecting the botnets on these devices. They can also circumvent formal rule-based detection systems. Although the machine learning-based system can detect the variances of the many kinds of attacks, the new distinct kinds of attacks can be launched sometimes. Additionally, the complex processing of ML classifiers is a challenge to implement the lightweight attack detection system on the resource constraint devices.

### 1.3. Our Contributions

In this study, our main contributions are as follows.

(1) A botnet attacks detection framework with sequential architecture based on machine learning (ML) algorithms is proposed for dealing with attacks in IoT environments.
(2) A correlated-feature selection approach is adopted for reducing the irrelevant features, which makes the system lightweight.
(3) In our proposal, classifiers based on different ML algorithms may be applied in different attack detection sub-engines, which leads to better detection performance and shorter processing times and a lightweight implementation.

### 1.4. Organization of the Paper

The rest of the paper is organized as follows: the related works of this study are included in Section 2, the background methodologies are shown in Section 3, the proposed detection architecture is explained in Section 4, and the detailed experiment results are expressed in Section 5. Finally, we conclude this study in Section 6.

## 2. Related Works

In recent years, many researchers are focusing more on the detection system for the IoT environment because the botnets attacks are targeting these devices. Network intrusion detection is one of the effective mechanisms to protect against malicious activities on the networks. Most of the public detection systems are based on the attacks’ signatures, which can be found as signature-based detection systems. These systems can detect known attacks by matching incoming attack patterns with the pre-recorded attack patterns. These systems cannot be lightweight because there are many signatures rules needed to be added in their databases. One of the popular detection systems, Snort [5], is also a signature-based system and uses attack signature rules to detect the cyber-attacks. They use a pattern-search algorithm, called Aho Corasick [7] to decide the incoming traffic pattern as attacks or not. Another detection system, Suricata [8], is a popular public IDS, fully supports multithreading architecture, and is more suitable for large-scale network systems. The study [9] used the Suricata to implement the detection system on the resource constraint device, Raspberry Pi. They aim to detect the port scanning attack on the IoT environment. The other studies [10,11] also proposed the attack detection system for the IoT environment, and they focused on port scanning, MITM, DNS cache poisoning, and flood attacks. The study [6] mentioned that Snort is lighter than Suricata. They also proposed the machine learning-based detection framework to extend the Snort system. Their results indicated that the detection results of their extension are better than the original Snort.

The study [12] proposed the multiple ANN-based attack detection method to improve the detection accuracy. Their experiments indicated that their proposed architecture could reduce the false-negative rate, even though it may increase the false-positive rate. The dataset, NSL-KDD, which was used in the experiments, is formally recognized for the traditional network attack detection system. The study [13] proposed a framework to select the effective machine learning algorithm for identifying malicious activities in IoT environments. They mentioned that naïve Bayes is an effective algorithm for the anomaly detection system in their experiments. The other study [14] proposed a cyber-attack detection system for sinkhole attacks that were targeting the IoT devices. They got the detection accuracy, 92% on fixed, and 72% on mobile scenarios with their study. The study [15] proposed the battery exhausted prevention system on the Bluetooth low energy (BLE), mask network. The other study [16] proposed a distributed denial-of-service (DDoS) attack detection mechanism on the big data situation. The studies [17,18] also proposed the entropy-based DDoS attack measurement on packet size interval and the method to locate the potential DDoS attack by the SDN network. Most of the studies mentioned above also tried to detect the cyber-attacks on IoT. However, these were mostly focusing on the detection system for specific types of threats targeting IoT environments.

The study [19] proposed the intrusion detection system for fog computing, and their proposal was based on the multilayer perceptron (MLP). Their experiments with two datasets [20,21] achieved the 94% accuracy with ADFA-LD and 74% accuracy with ADFA-WD. Basically, the MLP is a type of artificial neural network, and it is heavier than some other classifiers. Moreover, the new distinct kinds of attacks can be launched sometimes although the machine learning-based system can have reliable accuracy.

The preliminary of the sequential attack detection work [22] was also based on the machine learning methodology. An artificial neural network (ANN) architecture was applied to detect the botnets’ attacks. The experiment results indicated that the simple ANN architecture was enough to recognize the botnets’ attacks with this sequential architecture. We found that the detection performance is the best if the rectified linear unit (ReLU) function [23] was applied on hidden nodes, and the sigmoid function [23] was assigned on the output node. In this study, we extend our previous work with a feature selection method to reduce the high resource demand for further lightweight detection systems. We also propose the extensible botnet attacks detection framework, and we compare the evaluation results with different machine learning algorithms.

## 3. Background Methodologies

The network intrusion detection (NIDS) is one of the essential mechanisms to protect against malicious behavior on network-connected devices, like in an IoT environment. However, most of the detection systems are signature-based. It is difficult to detect new kinds of attacks even if these attacks are just the variances of the known attacks because these are used as the pre-recorded attacks’ signatures to identify the incoming network traffic pattern is an attack or not. That is, the machine learning-based techniques become the practical way to detect not only the known attacks but also their variances. In this study, we applied the popular machine learning methodologies, such as the artificial neural network (ANN), J48, and naïve Bayes. Moreover, the proposed system adopted the well-known feature selection algorithm, the correlation-based feature selection (CFS).

### 3.1. Artificial Neural Network (ANN)

The artificial neural network (ANN) is a basic structure of the neural network methodology. It is a computational model that is based on biological neural networks. However, it is a practical model for learning vector-valued functions [23]. The elements, also called neurons, are working in parallel, and these are connected with each other. A subgroup, dealing with the elements which are connected via the links, is called the layer. The layers basically include the input, hidden, and output layers. Each connecting link possesses the related weights, but these are needed to adjust to having optimal weight values by the backward calculation phases. Thus, the activation function becomes the critical role model to get these optimal weight vectors. The popular activations, such as sigmoid, ReLU, and tanh functions, are shown in Equations (1)–(3), respectively.

(1) $$f\left(x\right)=\frac{1}{1+e^{-x}}$$

(2) $$f\left(x\right)=\frac{\sin h\left(x\right)}{\cos h\left(x\right)}=\frac{e^{x}-e^{x-x}}{e^{x}+e^{-x}}$$

(3) $$f\left(x\right)=\max\left(0,x\right)=\{\begin{array}{ll} x, & x\geq 0\\ 0, & x<0 \end{array}$$

### 3.2. J48 Algorithm

J48, also called C4.5, is the descendant of the ID3 algorithm, which is developed by Ross Quinlan [24]. It is a tree-based algorithm that consists of the root node, and leave nodes are derived from the root. The tree is constructed by calculating the information gain and entropy values for all of the attribute values. It is a popular classification algorithm, and also it can handle uncompleted data points, continuous and discrete values. It can avoid the overfitting problem because of the pruning techniques.

### 3.3. Naïve Bayes

The naïve Bayes classifier provides features-learning, which is independent with the given class, and it represents using and learning probabilistic knowledge. It is also designed to be used in a supervised manner, like the decision tree algorithms. It is based on the Bayesian theorem and is particularly suited when the dimensionality of the input is high. It can often outperform a more sophisticated classification result, although its theory is simple. Moreover, the prediction time is independent of the number of instances, although the learning time is linear to the number of cases [24].

### 3.4. Correlation-Based Feature Selection

The feature selection process is for selecting the most important features or selecting the irrelevant features from all original features. Although the unnecessary features are removed, the features should offset the accuracy of a predictive model [25]. Although the filter-based features selection approaches are much lighter than the wrapper and embedded approaches, the selection method should match with the learning algorithms. The correlation-based feature selection algorithm is also a filter-based algorithm, and it bases on its own merit function. Its function is for evaluating the relations between input and output variables [26]. Though it is a simple technique, and it is efficient for evaluating the relations with the output variable and the uncorrelation with each feature. Thus, it cannot seriously affect the processing accuracy when it is dropped. This algorithm is claimed to be able to accomplish feature selection for classification on the correlation between features and classes. The studies [27,28] showed that the irrelevant features, redundant information also should be eliminated. The CFS algorithm is based on its merit function, and it can decide the best feature set that contains features highly correlated with the class, and also uncorrelated with each other. The original work of this approach can be found in [29]. Due to the lightweight processing purpose, the irrelevant or redundant features should be ignored. Basically, the many irrelevant features can also undesirably affect the processing accuracy. The best features set can be extracted with the highest merit that can compute it by using Equation (4). Where, *k* is the number of features in the current subset, $\bar{r_{cf}}$ is the average value of all “feature-class” correlations, and $\bar{r_{ff}}$ is the average value of all “feature-feature” correlations.

(4) $$M_{S_{k}}=\frac{k\bar{r_{cf}}}{\sqrt{k+k\left(k-1\right)\bar{r_{ff}}}}$$

## 4. Our Proposal: Sequential Attack Detection Architecture

**Figure 1:**
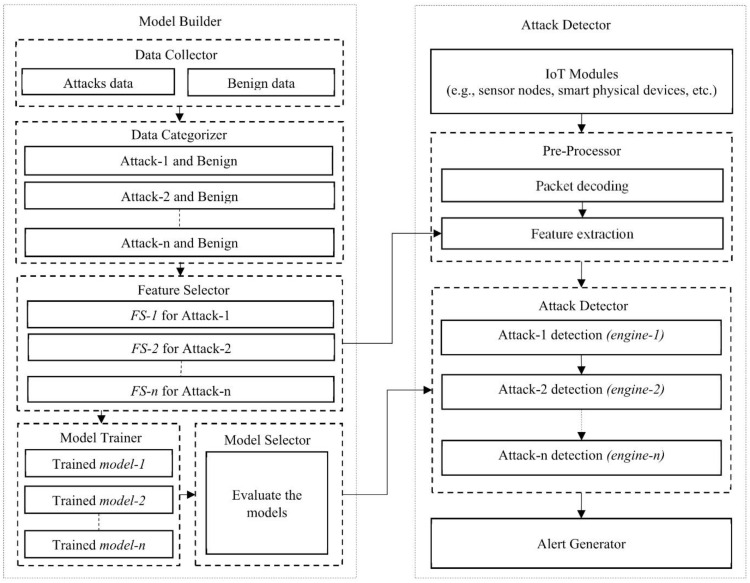
The block diagram of the proposed attack detection architecture.

There are mainly two phases in the proposed attack detection architecture, as shown in Figure 1. The first phase is the “Model Builder” to perform (i) data collection, (ii) data categorization, (iii) model training, and (iv) feature selection. In the “Data Collector,” it is needed to collect the benign data and the attacks data from the network, like in the IoT environment. The next module is “Data Categorizer”; it categorizes each kind of attack and benign data. Each category includes all data with the same attack class and benign data. There are eight categories in our experiments because of the eight different attack classes in the dataset. The module of “Feature Selector,” selects the most relevant features for each kind of attack class. This module is critical for implementing a lightweight detection system with high detection performance. The correlation-based feature selection approach will be adopted to select the relevant feature set for each kind of attack, like *FS-1*, *FS-2*, etc. After getting the most relevant feature sets, the trained model for each kind of attack will be made by the machine learning (ML) algorithms in the “Model Trainer” module. We tried several ML algorithms, including naïve Bayes, J48, and artificial neural network (ANN). As we mentioned [29], the filter-based feature selection methods are more matched with the ML-based detection systems, and the filter-based methods are lighter than other approaches, such as wrapper and embedded approaches. The “Model Selector” module implements and evaluates multiple different ML algorithms and selects the best one according to the detection accuracy for each of the sub-engines. In this way, different sub-engines may have different kinds of classifiers using different ML algorithms, which makes a hybrid detection architecture.

The module of “Attack Detector” is for detecting the attacks from the incoming network traffic. There are mainly two processes in “Pre-Processor,” packet decoding and feature extraction. Firstly, the incoming raw traffic patterns have to be decoded by preparing the script. It supports to get the feature data from the raw data. After getting the decoded traffic data, the features will be extracted using the same method as the above-mentioned feature selector module. The “Attack Detector” module will sequentially perform the attack detection processes. The extracted features will be passed through each of the sub-engines with a sequential manner to detect the attacks. If the incoming traffic pattern is identified as a malicious pattern on any sub-engine, the system will generate an alert.

## 5. Experimental Results

In the experiment, each related feature data is extracted from incoming all packet data, which is based on the defined features sets for each category. In this phase, a feature selection method is applied for the system to be lightweight. It also needs to extract the corresponding features from the input traffic pattern as a pre-processing step. After that, this extracted packet data is passed through each attack detection sub-engine by the sequential nature. The main phase is the attack detection sub-engines for the detection of different kinds of attacks in this proposed framework. The detection engine in this phase consists of multiple sub-engines, each of which will perform for detecting each kind of attack. The number of specific sub-engines is decided according to how many kinds of specific attacks have been stored in the training database. This experiment is mainly focused on detecting botnet attacks because most of IoT malware launched these kinds of attack [30,31]. In each of the attack detection sub-engines, any kind of machine learning algorithm can be used. If a specific sub-engine detects the malicious data, the system will generate the alert for performing future action. If it is identified as the benign, the incoming traffic patterns will be passed through to the dedicated sub-engines as sequential nature. Even this architecture is using several sub-engines to filter, and there is no need to add additional processing resources because the attack detection processes will perform separately. Our previous study [29] mentioned that the detection process would only need 8.75 s for around 5000 incoming/testing data; even the experiment environment was a resource constraint device, Raspberry Pi. This means that the system may need to take just 1.75 milliseconds to detect a malicious pattern in each of the specific sub-engines. Moreover, the system can extend the additional attack detection sub-engines without any complexity even although our experiments are done by the eight specific sub-engines to detect effectively for DDoS attacks that are launched by the IoT botnets.

### 5.1. Dataset

**Figure 2:**
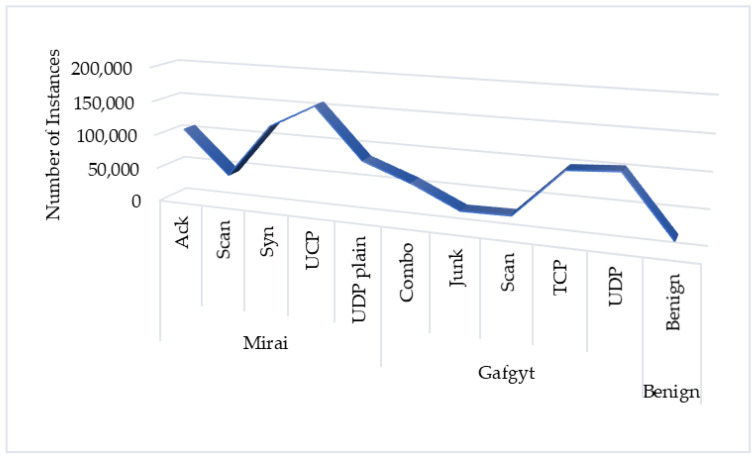
The distribution of the dataset.

These experiments are done using the dataset, N-BaIoT [32]. This dataset has a total of 849,234 instances, including 17,936 benign and 831,298 attack instances, which was captured by running the most dangerous IoT malware such as Mirai and BASHLITE [32,33]. ANN was applied for the experiments with the different well-known activation functions. This dataset is public and collected from an IoT environment, including popular IoT devices [32,33]. It has eight different classes of attacks based on two botnets and one benign and has 115 traffic features. The distribution of the instances in the dataset is shown in Figure 2.

The most well-known malware attacks by far on the IoT environments are Mirai and Gafgyt (BASHLITE). Once a device was infected, it automatically started scanning the network for new victims while waiting for instructions from the C & C server. The following lists are the sub-categories of these two attacks. There are five attacks included in the Gafgyt attacks, such as scan, junk, udp, tcp, and combo. Besides, there are three more attacks found in the Mirai attack, not only two kinds of the first botnet attacks (scan and udp), and also new kinds of three attacks (ack, syn, and udpplain). Some other variants of the malware have also launched attacks, like the following categories.

- Scan: Scanning the network for vulnerable devices.
- Junk: Sending spam data.
- Udp: UDP flooding.
- Tcp: TCP flooding.
- Combo: Sending spam data and opening a connection to a specified IP address and port.
- Ack: Ack flooding.
- Syn: Syn flooding.
- Udp plain: UDP flooding with fewer options, optimized for higher Packets Per Seconds (PPS).

Firstly, the dataset was reorganized by each attack data with benign data. For these categories, 66% of instances are selected as training data and the remaining part of the dataset as validating data. As the pre-processing step, we used the max–min normalization where the data inputs are mapped into the range between 0 and 1 [34]. We used some python libraries, such as Scikit-learn, Keras, and Tensorflow, to support the implementation of the system.

Scikit-learn: it is the supporting tool to implement many machine learning algorithms efficiently [35]. It also provides the function to split datasets into multiple subsets, including for splitting the training and test sets. We used this library to split the selected dataset into the training and testing data. In addition, we used this library to conduct experiments with the tree-based algorithm and naïve Bayes.

Keras: it is a high-level neural network Python-API, and capable of running on top of the dependencies, including TensorFlow. It was developed with a focus on enabling fast experimentation [36]. It is running on the top of the Tensorflow [37]. It can also support multilayer perception.

Tensorflow: it is the more complex library for distributed numerical computation using data flow graphs. It is possible to train and run extensive neural networks efficiently [35]. It was created at Google, and it is an open-source software library for high-performance numerical computation [38]. It can strongly support machine learning and deep learning for many other scientific domains.

**Figure 3:**
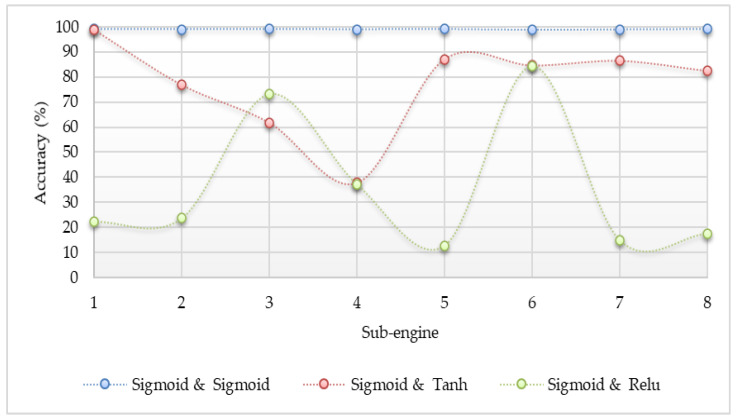
Accuracy comparison for detecting Internet of Things (IoT) botnet attacks by a simple neural network (sigmoid in the hidden layer and different functions in the output layer).

**Figure 4:**
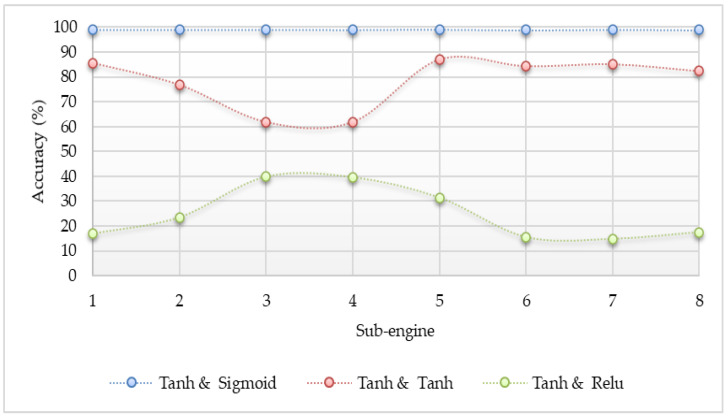
Accuracy comparison for detecting IoT botnets attacks by a simple neural network (tanh in the hidden layer and different functions in the output layer).

**Figure 5:**
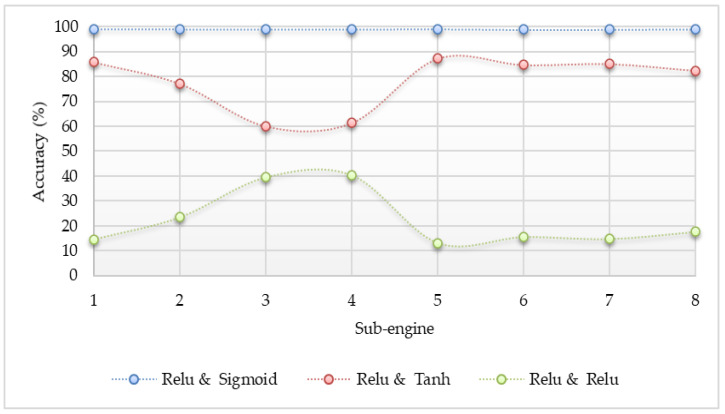
Accuracy comparison for detecting IoT botnets attacks by a simple neural network (ReLU in the hidden layer and different functions in the output layer).

The different activation functions such as sigmoid, Tanh, and ReLU were applied to determine which one is the best option in the proposed system. The accuracy result comparisons are shown in Figure 3, Figure 4 and Figure 5. The accuracy in the experiment was calculated by Equation (5). Where, “TP” is true positive which indicates the number of attack classes correctly classified, “TN” is true negative which indicates the number of benign classes correctly classified, “FP” is false positive which indicates the number of benign classes wrongly classified as an attack, and “FN” is false negative which indicates the number of attack class wrongly classified as benign. According to the experiment results, the sigmoid function gave the best accuracy results.

(5) $$Accuracy=\frac{TP+TN}{TP+TN+FP+FN}$$

### 5.2. The Performance with ANN

The detection results for the Mirai and Gafgyt attacks using a simple artificial neural network are shown in Figure 3, Figure 4 and Figure 5, respectively. In the hidden and output layer, sigmoid or tanh or ReLU function is applied. There are eight sub-engines included in this experiment by representing 1 for ack, 2 for combo, 3 for junk, 4 for scan, 5 for syn, 6 for tcp, 7 for udp, and 8 for udpplain. As the pre-processing step, we applied the max–min normalization, where the data inputs are mapped into the range between 0 and 1 [34]. The max–min normalization approach is shown in Equation (6).

(6) $$z=\frac{x-\min\left(x\right)}{\max\left(x\right)-\min\left(x\right)}$$

The experiment results indicate that the detection performance is the best if the detection model used the sigmoid activation function for the output layer. All of the detection accuracy using the sigmoid action function at the output layer is up to 99%. Although the configuration of the Tanh activation function at the output layer is better than the configuration of the ReLU activation function, some of the results are not stable and are lower than the configuration of the sigmoid action function at the hidden layer. Even though the activation functions were changed at the hidden layer, it can be observed that the output has no clear difference from each other. According to the results, the configuration of the sigmoid activation function at the output layer yielded the most accurate results, and it could apply any activation functions at the hidden layer without sacrificing detection accuracy. However, the ReLU activation function will be applied for the hidden layer because this function is lighter than the other two, and it does not need the calculation of the exponential values.

### 5.3. Comparison with Different Learning Algorithms

Firstly, we evaluated the performance of our proposed detection architecture by removing the feature selector module. The detection results of ANN’s and other algorithms are compared in Table 1. According to the evaluation mentioned above results (in Figure 3, Figure 4 and Figure 5) between different activation on ANN, the performance comparisons were completed with ReLU function in the hidden layer and sigmoid function in the output layer because it is the best choice for the system to be lightweight and more accurate. The Naïve Bayes (NB) supports the 85.81 for ack, 77.33 for combo, 61.52 for junk, 76.11 for scan, 87.08 for syn, 84.57 for tcp, 85.18 for udp, and 82.49 for udpplain attacks, respectively. The detection results are not too good, especially in junk attack detection. However, the detection results of J48 and simple ANN are significantly better than that of NB, and also these are good enough for detecting the different kinds of IoT botnets attacks. The J48 results are 99.01 for ack, 99.01 for combo, 99.08 for junk, 99.05 for scan, 99.08 for syn, 99.05 for tcp, 98.99 for udp, and 99.09 for udpplain attacks. The ANN results are also high enough, such as 99.01 for ack, 99 for junk, 98.98 for scan, 99.01 for syn, 98.95 for tcp, 98.97 for udp, and 99 for udpplain attacks. The results show that ANN (ReLU in the hidden layer and sigmoid in the output layer) and J48 are significantly better than the NB algorithm.

The performance results of the proposed attack detection architecture using the feature selector module are shown in Table 2. In this architecture, the feature selection method is adopted to reduce the irrelevant features from the dataset. We also evaluated our detection architecture by NB, J48, and ANN. The feature selection processes were completed by the best merit values of each class; 0.996 for ack, 0.995 for combo, 0.994 for junk, 0.99 for scan, 0.996 for syn, 0.995 for tcp, 0.995 for udp, and 0.996 for udpplain attacks. The detection results show that there are no differences between using all features and using selected features with J48 and ANN even significantly reduced the number of features from the original 115 features.

Moreover, the results of selected features with NB are significantly better than the results using all features, and these results can be comparable with the results of the other two algorithms, J48 and ANN. With the proposed detection architecture, the detection results of NB reached up to 99.09 for ack, 99.09 for combo, 99.1 for junk, 97.34 for scan, 99.1 for syn, 98.99 for tcp, 98.97 for udp, and 99.1 for udpplain, respectively. According to the performance results with different three algorithms, our proposed detection is suitable for detection of botnets attacks in the IoT environment. Moreover, our proposed architecture has no limit to extend additional detection engines for future challengeable attacks.

### 5.4. Performance of the Proposed Detection Architecture

**Figure 6:**
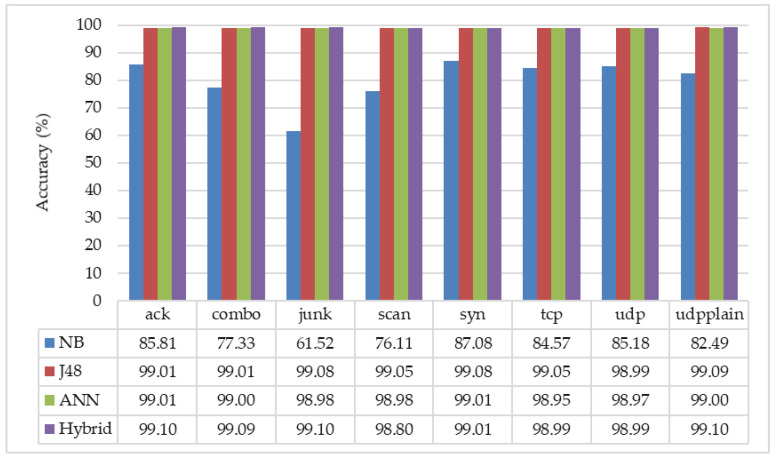
Comparison of our proposed sequential detection scheme (hybrid classifier) with the formal detection scheme (naïve Bayes (NB), J48, and artificial neural network (ANN)).

We compared the detection accuracy between the formal detection scheme using different classifiers, and our proposed detection scheme with a hybrid classification is shown in Figure 6. For the formal detection scheme, we used different machine learning algorithms, such as NB, J48, or ANN. In this detection scheme, a machine learning algorithm is needed to classify whether the incoming pattern is an attack or not. In our scheme (described in Figure 1), we proposed the sequential detection architecture with different attack detection engines with the “hybrid classification.” Each of the sub-engines need to be assigned with the most suitable classifier, and the selected classifiers can be different in each engine to have the best detection performance. For this purpose, we found the most suitable classifier for each sub-engine by comparing the accuracy values (which are based on Equation (5)).

The proposed scheme performs the four processes, such as feature selector, model trainer, and model selector modules to achieve the accuracy values. Firstly, we applied the feature selector module for recognizing the most relevant features for every attack detection, “sub-engines.” We adopted the CFS to select the most important features sets for each of the sub-engines, by deciding with its own merit function. After that, we constructed the attack detecting models by training with different classifiers, such as NB, J48, and simple ANN. After getting the trained models, we evaluated these models to determine which classifier can give the most accurate results. If the two classifiers could perform with the same detection accuracy, the faster detection classifier was applied to our proposed scheme. That is, our proposed scheme can detect malicious patterns effectively with lightweight architecture.

The evaluation results of different classifiers using the feature selector are described in Table 2. According to the evaluation results, we assigned the different classifiers as “hybrid classification” in different sub-engines, i.e., J48 in *sub-engine-1*, NB in *sub-engine-2 and 3*, J48 in *sub-engine-4*, NB in *sub-engine-5 and 6*, J48 in *sub-engine-7*, and NB in *sub-engine-8*, respectively. The results in Figure 6 show that our detection scheme is significantly better than the formal detection architecture with NB and ANN classifiers. Moreover, the results with our detection scheme are better than the formal detection scheme with J48, especially in ack, combo, junk, and udpplain. Additionally, the processing time of our detection scheme is shorter than the formal detection scheme, and the processing time comparison is shown in Table 3. It just needs 0.03 s to perform the 270,908 samples in the attack detector module.

The processing time comparison of our proposed scheme with the formal detection scheme is shown in Table 3. Our experiments were done on a personal computer which possesses i7-8565U and 16 GB RAM. We separated two-thirds of the dataset for the model trainer, and the remaining part was for model evaluation. The above-mentioned results in the table are expressed by the processing time of the model trainer module for “Training”, and the attack detector module for “Testing”. In the formal detection architecture, the model training time of NB and J48 is not too different, but these classifiers are lighter than the other classifier, ANN. Moreover, the processing time of attack detection with J48 is faster than the other two classifiers in the formal architecture. In addition, the processing time of our proposed architecture with the “hybrid” classification is faster than the formal detection architecture, in the cases of NB, J48, and ANN. In the proposed architecture, the feature selector module could greatly reduce the number of the original features. Moreover, the model selector module could support choosing the classifiers with the best performance, which are based on the better detection accuracy and the faster processing time for each of sub-engines. Therefore, our proposed scheme with “hybrid” classification is lighter and has better accuracy than the formal detection architecture. We also tried the model trainer and attack detector modules with sub-engines in parallel. In parallel training, the processing time of the model trainer module is three times faster than the serial training. Additionally, the processing of the attack detection module is slightly faster than the serial classification, but the running environment is needed to support the multithreading architecture. The experiment results indicate that our proposed scheme is lightweight and has better accuracy for the detection of IoT botnets attacks with sequential architecture.

### 5.5. Observations

According to the experimental results, it can be observed that:

- The proposed architecture, sequential detection scheme with “hybrid” classification, is useful to detect the IoT botnets attacks. In Figure 6, the average detection accuracy of “hybrid” classification is around 99% in each of the sub-engines.
- In the construction of neural network architecture, the sigmoid function at the output layer can generate the most accurate results by our detection scheme. Even though any activation functions can be used at the hidden layer without significantly affecting detection accuracy, the implementation of the ReLU activation function at the hidden layer is more suitable because this function is lighter than the other two functions.
- The above-mentioned results show that J48 and NB are lighter than ANN even if the minimum configuration is done with a single hidden layer and a single output node. Moreover, the detection accuracy of these three classifiers is almost the same in our proposed detection scheme with the feature selector. Due to the feature selector module, the system got more accurate results and the lighter processing capability. Especially in the NB classifier, the detection accuracy for junk attack is dramatically increased up to 99.10% from 61.52%.
- Due to the model selector module, we could assign the most suitable classifier as “hybrid classification” in each of the sub-engines. It supports the system to have the most accurate results among different classifiers.
- Our detection can also be extended with additional sub-engines if necessary.

## 6. Conclusions and Future Work

With the rapid development the IoT technologies, the cyber-attacks are mostly targeting these devices. Especially, the botnets attacks are vastly challenging in these environments. After infecting the botnets, the attackers can control these devices via the C & C server and launch attacks to the victim hosts. Our proposed detection architecture can detect the known attacks and their variances, and also, the system can be extended to detect the new kinds of upcoming attacks. Moreover, the system achieved the lightweight nature and the best detection accuracy by applying the feature selection approach. The hybrid classification could also greatly assist with achieving the best accuracy with rapid detection. In our future work, we will investigate the normal traffic patterns on the different natures of IoT devices to extend the anomaly-sub-engine for detecting unknown attacks effectively. Moreover, we will implement a testbed to validate the performance of our proposed system, and propose how to isolate the compromised devices.

## Figures and Tables

**Table 1:** Detection accuracy without feature selector.

Classifier	Ack	Combo	Junk	Scan	Syn	Tcp	Udp	Udpplain
NB	85.81	77.33	61.52	76.11	87.08	84.57	85.18	82.49
J48	99.01	99.01	99.08	99.05	99.08	99.05	98.99	99.09
ANN	99.01	99.00	98.98	98.98	99.01	98.95	98.97	99.00

**Table 2:** Detection accuracy with feature selector.

Classifier	Ack	Combo	Junk	Scan	Syn	Tcp	Udp	Udpplain
NB	99.09	99.09	99.10	97.34	99.01	98.99	98.97	99.10
J48	99.10	99.07	99.08	98.80	98.99	98.98	98.99	99.09
ANN	99.01	98.98	98.99	98.71	98.90	98.89	98.90	99.00

**Table 3:** Processing time comparison (seconds).

Classifier	Training	Testing
NB	16.06	0.44
J48	15.05	0.07
ANN	151.32	9.71
Hybrid (serial)	1.56	0.03
Hybrid (parallel)	0.46	0.02
